# Lung metastases at the initial diagnosis of high-grade osteosarcoma: prevalence, risk factors and prognostic factors. A large population-based cohort study

**DOI:** 10.1590/1516-3180.2018.0381120619

**Published:** 2020-01-13

**Authors:** Chao Zhang, Xu Guo, Yao Xu, Xiuxin Han, Jun Cai, Xin Wang, Guowen Wang

**Affiliations:** I MD, MSc. Surgeon, Department of Bone and Soft Tissue Tumors, Tianjin Medical University Cancer Institute and Hospital, National Clinical Research Center for Cancer, Key Laboratory of Cancer Prevention and Therapy, Tianjin’s Clinical Research Center for Cancer, Tianjin, China.; II MD, MSc. Surgeon, Department of Bone and Soft Tissue Tumors, Tianjin Medical University Cancer Institute and Hospital, National Clinical Research Center for Cancer, Key Laboratory of Cancer Prevention and Therapy, Tianjin’s Clinical Research Center for Cancer, Tianjin, China.; III MBBS. Doctoral Student, Department of Bone and Soft Tissue Tumors, Tianjin Medical University Cancer Institute and Hospital, National Clinical Research Center for Cancer, Key Laboratory of Cancer Prevention and Therapy, Tianjin’s Clinical Research Center for Cancer, Tianjin, China.; IV MD, MSc. Surgeon, Department of Bone and Soft Tissue Tumors, Tianjin Medical University Cancer Institute and Hospital, National Clinical Research Center for Cancer, Key Laboratory of Cancer Prevention and Therapy, Tianjin’s Clinical Research Center for Cancer, Tianjin, China.; V PhD. Assistant Professor, Centre for Research and Development of Anti-Tumor Drugs, Tianjin Institute of Medical and Pharmaceutical Sciences, Tianjin, China.; VI PhD. Associate Professor, Department of Epidemiology and Biostatistics, First Affiliated Hospital, Army Medical University, Chongqing, China.; VII MD, MSc. Surgeon, Department of Bone and Soft Tissue Tumors, Tianjin Medical University Cancer Institute and Hospital, National Clinical Research Center for Cancer, Key Laboratory of Cancer Prevention and Therapy, Tianjin’s Clinical Research Center for Cancer, Tianjin, China.

**Keywords:** Survival analysis, Osteosarcoma, Neoplasm metastasis, Retrospective studies

## Abstract

**BACKGROUND:**

Osteosarcoma is the most prevalent malignant bone tumor in children and adolescents. Lung metastases are associated with poor prognosis.

**OBJECTIVE:**

The aim here was to explore the prevalence of and risk and prognostic factors for lung metastases in high-grade osteosarcoma patients.

**DESIGN AND SETTING:**

Retrospective cohort study based on the Surveillance, Epidemiology and End Results (SEER) database in the United States.

**METHODS:**

Data on 1,408 high-grade osteosarcoma patients registered in the SEER database between 2010 and 2015 were extracted. From these, all patients with high-grade osteosarcoma and initial lung metastasis were selected for analysis on risk and prognostic factors for lung metastases. Overall survival was estimated.

**RESULTS:**

There were 238 patients (16.90%) with lung metastases at diagnosis. Axial location, tumor size > 10 cm (odds ratio, OR 3.19; 95% confidence interval, CI: 1.58-6.45), higher N stage (OR 4.84; 95% CI: 1.94-12.13) and presence of bone metastases (OR 8.73; 95% CI: 4.37-17.48) or brain metastases (OR 25.63; 95% CI: 1.55-422.86) were significantly associated with lung metastases. Younger age and surgical treatment (hazard ratio, HR 0.46; 95% CI: 0.30-0.71) favored survival. Median survival was prolonged through primary tumor surgery.

**CONCLUSIONS:**

The factors revealed here may guide lung metastasis screening and prophylactic treatment for osteosarcoma patients. A primary tumor in an axial location, greater primary tumor size, higher lymph node stage and presence of bone or brain metastases were significantly correlated with lung metastases. The elderly group (≥ 60 years) showed significant correlation with poor overall survival. For improved survival among high-grade osteosarcoma patients with lung metastases, aggressive surgery on the primary tumor site should be encouraged.

## INTRODUCTION

Osteosarcoma is the most prevalent malignant bone tumor in children and adolescents. An overall annual prevalence of 0.2-3 cases per 100,000 population has been reported.^1^ Despite the rarity of osteosarcoma, it remains one of the deadliest cancers during the pubertal growth spurt. Lung metastases have been reported to be one of the challenging factors associated with a poor prognosis.^2^

Approximately 20% of osteosarcoma patients present with metastatic disease at the time of the initial diagnosis. The most prevalent metastatic type is lung metastasis, which occurs in more than 80% of the cases.^3^^,^^4^ Despite the development of novel treatments for osteosarcoma, 30-40% of these patients still relapse and the long-term post-relapse survival among these individuals has been reported to be less than 20%.^5^^,^^6^

Undoubtedly, osteosarcoma patients can benefit from early diagnosis and treatment of metastases. Thus, a reasonable degree of lung metastasis screening for osteosarcoma patients at diagnosis is important. Male sex and the site involved (femur and tibia) were confirmed to be associate with greater occurrence of metastasis in a Mexican clinical trial.^7^ The primary tumor size was reported to be a risk factor for lung metastasis among patients with osteosarcoma.^8^^,^^9^

Currently, radiography is one of the most widely applied clinical screening strategies. However, radiography barely captures metastases until they physically form. Therefore, studies looking into the risk factors for lung metastasis occurrence among patients with osteosarcoma are warranted.

The prognostic factors for osteosarcoma patients with lung metastases have been drawing the attention of researchers around the world. In previous studies, a series of prognostic factors for lung metastases in osteosarcoma patients were reported, including sex,^10^ non-necrotic metastases,^11^ number of pulmonary nodules,^3^^,^^5^^-^^6^ late relapse,^12^^,^^13^ unilateral lung involvement,^6^^,^^12^ completeness of surgical resection of all tumor sites detected^5^^,^^6^ and histological response to chemotherapy.^13^ There is a need for studies on large populations, to analyze the prognostic factors for lung metastases among osteosarcoma patients. Furthermore, there is a need to investigate factors associated with survival among patients with high-grade osteosarcoma with lung metastases.

## OBJECTIVE

In the present study, in which information from the Surveillance, Epidemiology, and End Results (SEER) database was analyzed, we aimed to investigate the prevalence of and risk factors for lung metastases among high-grade osteosarcoma patients. Furthermore, survival analysis was conducted to evaluate the prognostic factors for high-grade osteosarcoma with lung metastases.

## METHODS

### Study design, ethics and setting

This was a retrospective cohort study based on a database of patients with cancer in the United States. This study complied with the 1964 Helsinki Declaration and its later amendments or comparable ethical standards. The study protocol was approved by the Tianjin Medical University Cancer Institute & Hospital Ethical Review Board (Ek2018022).

### Study population

The National Cancer Institute’s open public database, Surveillance, Epidemiology and End Results (SEER), provides cancer incidence and survival data from 18 established cancer registries across the United States. SEER is a particularly useful tool for assessing the epidemiological characteristics of cancer.

Data on cancer patients were obtained from the SEER database. The SEER*Stat 8.3.5 software (https://seer.cancer.gov/data/) was used to generate the case listing. Since the details of metastases were not available before 2010, data on high-grade osteosarcoma patients diagnosed between 2010 and 2015 were collected. Patients diagnosed as having “9192/3: parosteal osteosarcoma” or “9187/3: intraosseous well-differentiated osteosarcoma” were excluded from the analyses, given that these diagnoses present a less aggressive clinical course, compared with other high-grade subtypes. Such patients are treated differently: they are diagnosed at autopsy or via the death certificate, and any presence of lung metastases or implementation of follow-up remains unknown.^14^

From this database, 1,408 patients who had been identified as presenting high-grade osteosarcoma between January 1, 2010, and December 31, 2015, were selected for analysis on the prevalence of lung metastases and their risk factors. Data on those diagnosed as presenting high-grade osteosarcoma with lung metastases between 2010 and 2014 (i.e. with at least one year of follow-up) were used to conduct survival analysis and to investigate the prognostic factors for lung metastases.

### Variables and statistical analysis

The patients’ demographic and clinical characteristics were included and categorized as follows: age (≤ 24, 25-59 or ≥ 60 years); sex (female or male); race [white, black, AI (American Indian/Alaska Native) or API (Asian or Pacific Islander)]; marital status (married or unmarried); insurance status (insured or uninsured); location (extremities: long and short bones of the upper and lower extremities; axial skeleton: pelvis, spine and ribs; or others: mandible, skull and other atypical locations); tumor size (≤ 5, 5-10 or > 10 cm); regional lymph node stage (N0 or N1); histology (osteosarcoma and not otherwise specified (NOS) or others); and presence or absence of bone metastases, liver metastases or brain metastases.

The differences in prevalence of lung metastases between the categorical variables were analyzed using Pearson’s chi-square test or the rank-sum test. The risk factors for high-grade osteosarcoma patients with initial lung metastases were determined primarily through univariable logistic regression. Moreover, factors that achieved significant levels were incorporated into the multivariable logistic regression model to control the potential confounding factors.

The primary outcome from the survival analysis was the overall survival, which was defined as the length of time from when the high-grade osteosarcoma was first diagnosed to the occurrence of all causes of death. Kaplan-Meier curves and log-rank tests were used to analyze survival differences. At the same time, multivariable Cox proportional-hazards regression was conducted based on the aforementioned factors, with P-values < 0.05 taken to be significant, and taking into account the surgical treatments applied to the primary site (not applied or applied).

All statistical analyses were performed using the Statistical Package for the Social Sciences (SPSS) 23.0 (IBM Corporation, Armonk, NY, USA) and all charts on survival were produced using MedCalc 15.2.2. Two-tailed P < 0.05 was considered to be statistically significant.

## RESULTS

### Demographic and clinical characteristics

**Figure 1.**
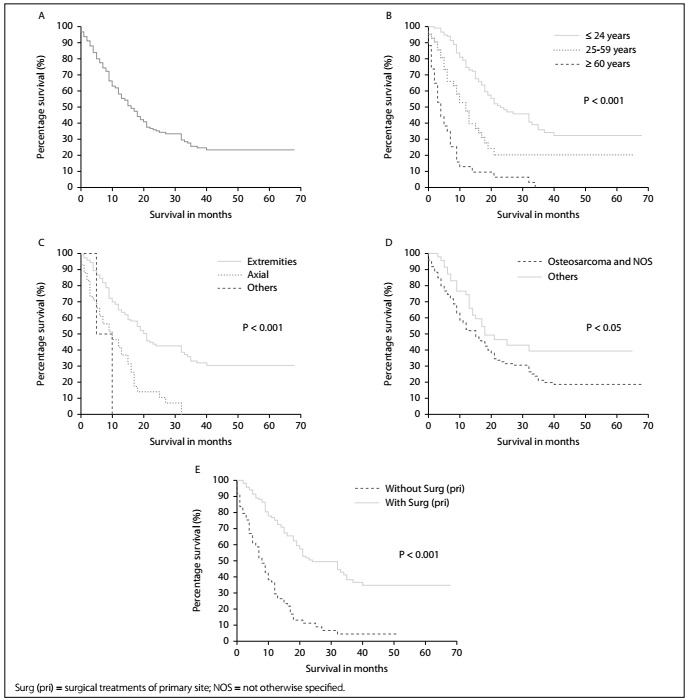
Kaplan-Meier analyses on overall survival among high-grade osteosarcoma patients. The high-grade osteosarcoma patients with lung metastases are shown together (A, overall) and stratified according to age (B), location (C), histology (D) and surgical treatments of the primary site (E).

A total of 1,408 high-grade osteosarcoma patients were included in the current study. Among them, 623 (44.2%) were male and 785 were female (55.8%). Their mean age was 29.95 ± 22.10 years. Most of the participants involved were white (N = 1,056; 75.0%). Among the participants, there were 195 high-grade osteosarcoma patients with lung metastases [69 males (35.4%) and 126 females (64.6%); mean age of 30.16 ± 24.09 years] who had been followed up for at least one year (Figure 1, Table 1).

**Table 1.** Baseline of the demographic and related clinical characteristics among patients diagnosed with high-grade osteosarcoma with and without lung metastases (LM)

Subject characteristics	Number of osteosarcoma patients (2010-2015)		P-value	Number of osteosarcoma patients (2010-2014)		P-value
	With LM (n, %)	Without LM (n, %)		With LM (n, %)	Without LM (n, %)	
Age, in years						
≤ 24	147 (17.78)	680 (82.22)	0.02	119 (17.40)	565 (82.60)	0.09
25-59	48 (12.66)	331 (87.34)		42 (13.46)	270 (86.54)	
≥ 60	43 (21.29)	159 (78.71)		34 (21.12)	127 (78.88)	
Sex						
Female	92 (14.77)	531 (85.23)	0.06	69 (13.48)	443 (86.52)	0.01
Male	146 (18.60)	639 (81.40)		126 (19.53)	519 (80.47)	
Race						
White	176 (16.67)	880 (83.33)	0.92	145 (16.67)	725 (83.33)	0.90
Black	40 (18.02)	182 (81.98)		31 (17.42)	147 (82.58)	
AI	3 (21.43)	11 (78.57)		3 (23.08)	10 (76.92)	
API	18 (16.07)	94 (83.93)		15 (16.13)	78 (83.87)	
Unknown	1 (25.00)	3 (75.00)		1 (33.33)	2 (66.67)	
Marital status						
Unmarried	181 (17.22)	870 (82.78)	0.34	150 (17.30)	717 (82.70)	0.33
Married	49 (14.98)	278 (85.02)		38 (14.56)	223 (85.44)	
Unknown	8 (26.67)	22 (73.33)		7 (24.14)	21 (75.86)	
Insurance status						
Insured	228 (17.09)	1,106 (82.91)	0.74	185 (16.88)	911 (83.12)	0.91
Uninsured	7 (15.22)	39 (84.78)		7 (17.95)	32 (82.05)	
Unknown	3 (10.71)	25 (89.29)		3 (13.64)	19 (86.36)	
Location						
Extremities	174 (17.42)	825 (82.58)	< 0.001	144 (17.50)	679 (82.50)	< 0.001
Axial	54 (23.08)	180 (76.92)		42 (22.58)	144 (77.42)	
Others	2 (1.28)	154 (98.72)		2 (1.50)	131 (98.50)	
Unknown	8 (42.11)	11 (57.89)		7 (46.67)	8 (53.33)	
Tumor size (cm)						
≤ 5	12 (5.63)	201 (94.37)	< 0.001	12 (6.63)	169 (93.37)	< 0.001
5-10	69 (13.22)	453 (86.78)		57 (13.60)	362 (86.40)	
> 10	108 (22.78)	366 (77.22)		87 (22.54)	299 (77.46)	
Unknown	49 (24.62)	150 (75.38)		39 (22.81)	132 (77.19)	
N stage						
N0	199 (15.06)	1,122 (84.94)	< 0.001	163 (15.02)	922 (84.98)	< 0.001
N1	16 (50.00)	16 (50.00)		13 (54.17)	11 (45.83)	
Unknown	23 (41.82)	32 (58.18)		19 (39.58)	29 (60.42)	
Histology						
Osteosarcoma and NOS	179 (18.19)	805 (81.81)	0.05	147 (18.26)	658 (81.74)	0.05
Others	59 (13.92)	365 (86.08)		48 (13.64)	304 (86.36)	
Bone metastasis						
None	193 (14.41)	1,146 (85.59)	< 0.001	158 (14.39)	940 (85.61)	< 0.001
Yes	39 (63.93)	22 (36.07)		31 (60.78)	20 (39.22)	
Unknown	6 (75.00)	2 (25.00)		6 (75.00)	2 (25.00)	
Liver metastasis						
None	230 (16.49)	1,165 (83.51)	0.03	188 (16.42)	957 (83.58)	< 0.001
Yes	4 (44.44)	5 (55.56)		3 (37.50)	5 (62.50)	
Unknown	4 (100.00)	0 (0.00)		4 (100.00)	0 (0.00)	
Brain metastasis						
None	229 (16.39)	1,168 (83.61)	0.001	186 (16.23)	960 (83.77)	< 0.001
Yes	4 (66.67)	2 (33.33)		4 (66.67)	2 (33.33)	
Unknown	5 (100.00)	0 (0.00)		5 (100.00)	0 (0.00)	
Surg (pri)						
None	91 (37.60)	151 (62.40)	< 0.001	74 (36.27)	130 (63.73)	< 0.001
Yes	147 (12.65)	1,015 (87.35)		121 (12.74)	829 (87.26)	
Unknown	0 (0.00)	4 (100.00)		0 (0.00)	3 (100.00)	
Total	238 (16.90)	1,170 (83.10)		195 (16.85)	962 (83.15)	

LM = lung metastases; AI = American Indian/Alaska Native; API = Asian or Pacific Islander; NOS = not otherwise specified; Surg (pri) = surgical treatments of primary site.

### Prevalence of lung metastases

Among the 1,408 eligible patients with high-grade osteosarcoma, an initial lung metastasis was diagnosed in 16.90% of the entire cohort (238/1,408). Patients in the age group ≥ 60 years presented significantly higher prevalence of lung metastases than did the younger age groups (χ^2^ = 8.05; P = 0.018). The prevalences of lung metastases in males and females were 14.77% and 18.60%, respectively, without any significant difference (χ^2^ = 3.63; P = 0.057). Moreover, the prevalence of lung metastases did not show any significant difference with regard to different racial groups (χ^2^ = 0.50; P = 0.92) (Table 1).

### Risk factors for developing lung metastases

Univariable analysis showed that the factors of axial location (odds ratio, OR = 1.42; 95% confidence interval, CI = 1.01-2.01; P = 0.045), greater primary tumor size (OR = 4.94; 95% CI = 1.01-2.01; P < 0.001), higher regional lymph node (N) stage (OR = 5.64; 95% CI = 2.77-11.46; P < 0.001), presence of bone metastasis (OR = 10.53; 95% CI = 6.11-18.14; P < 0.001), presence of liver metastasis (OR = 4.05; 95% CI = 1.08-15.21, P = 0.04) and presence of brain metastasis (OR = 10.20; 95% CI = 1.86-56.02; P = 0.01) were positively associated with presence of lung metastases.

Multivariable logistic regression suggested that axial location, greater tumor size, higher N stage and presence of bone metastases or brain metastases were all significantly associated with lung metastases seen at the initial diagnosis (Table 2).

**Table 2.** Univariable and multivariable logistic regression for analysis on the associated factors for development of lung metastases among patients diagnosed with high-grade osteosarcoma (diagnosed between 2010 and 2015)

Subject characteristics	Univariable		Multivariable	
	OR (95% CI)	P-value	OR (95% CI)	P-value
Location				
Extremities	1 (Reference)	1.00	1 (Reference)	1.00
Axial	1.42 (1.01-2.01)	0.045	1.15 (0.72-1.83)	0.56
Others	0.06 (0.02-0.25)	< 0.001	0.04 (0.00-0.37)	0.01
Unknown	NA	NA	NA	NA
Tumor size (cm)				
≤ 5	1 (Reference)	1.00	1 (Reference)	1.00
5-10	2.55 (1.35-4.82)	0.004	1.73 (0.84-3.56)	0.14
> 10	4.94 (2.66-9.20)	< 0.001	3.19 (1.58-6.45)	0.001
Unknown	NA	NA	NA	NA
N stage				
N0	1 (Reference)	1.00	1 (Reference)	1.00
N1	5.64 (2.77-11.46)	< 0.001	4.84 (1.94-12.13)	0.001
Unknown	NA	NA	NA	NA
Bone metastasis				
None	1 (Reference)	1.00	1 (Reference)	1.00
Yes	10.53 (6.11-18.14)	< 0.001	8.73 (4.37-17.48)	< 0.001
Unknown	NA	NA	NA	NA
Liver metastasis				
None	1 (Reference)	1.00	1 (Reference)	1.00
Yes	4.05 (1.08-15.21)	0.04	0.00 (0.00- NA)	1.00
Unknown	NA	NA	NA	NA
Brain metastasis				
None	1 (Reference)	1.00	1 (Reference)	1.00
Yes	10.20 (1.86-56.02)	0.01	25.63 (1.55-422.86)	0.02
Unknown	NA	NA	NA	NA

OR = odds ratio; CI = confidence interval; NA = not available.

### Survival analysis and prognostic factors for lung metastases

Among the 195 high-grade osteosarcoma patients with lung metastases included in the one-year survival analysis, the end of the follow-up was marked by death in the cases of 128 of the patients (65.64%). The median overall survival time was 16.00 months (95% CI = 12.81-19.19 months; Figure 1A). Kaplan-Meier analysis showed the overall survival among subjects with older age (Figure 1B; P < 0.001), axial location (Figure 1C; P < 0.001) and osteosarcoma and NOS (Figure 1D; P < 0.05) was lower than that of their counterparts. Conversely, patients with surgical treatment of the primary site presented markedly higher overall survival than did the subjects without surgery (Figure 1E; P < 0.001).

In the multivariable Cox regression model, the results showed that elderly patients (≥ 60 years; hazard ratio, HR = 3.48; 95% CI = 2.14-5.66; P < 0.001) were associated with poor overall survival, with a median survival time of four months. However, surgery at the primary site was positively associated with better overall survival (HR = 0.46; 95% CI = 0.30-0.71; P < 0.001). The median survival time could be prolonged from 8 months to 24 months through surgery at the primary site (Table 3).

**Table 3.** Multivariable Cox regression for analysis on the prognostic factors for high-grade osteosarcoma with lung metastases (diagnosed between 2010 and 2014)

Subject characteristics	No. of osteosarcoma patients with LM		Survival, Median (IQR), mo	HR (95% CI)	P-value
	Overall	Deceased (n, %)			
Age, years					
≤ 24	119	65 (54.62)	23.00 (15.02-30.98)	1 (Reference)	1.00
25-59	42	30 (71.43)	12.00 (8.63-15.37)	1.87 (1.17-2.99)	0.01
≥ 60	34	33 (97.06)	4.00 (2.38-5.62)	3.48 (2.14-5.66)	< 0.001
Location					
Extremities	144	84 (58.33)	21.00 (17.09-24.91)	1 (Reference)	1.00
Axial	42	37 (88.10)	10.00 (4.80-15.20)	1.32 (0.83-2.10)	0.24
Others	2	2 (100.0)	5 (NA)	3.96 (0.93-16.81)	0.06
Unknown	7	5 (71.43)	NA	NA	NA
Histology					
Osteosarcoma and NOS	147	102 (69.39)	15.00 (11.03-18.97)	1 (Reference)	1.00
Others	48	26 (54.17)	18.00 (8.89-27.11)	0.68 (0.44-1.05)	0.08
Surg (pri)					
None	74	64 (86.49)	8.00 (5.82-10.19)	1 (Reference)	1.00
Yes	121	64 (52.89)	24.00 (14.89-33.11)	0.46 (0.30-0.71)	< 0.001

LM = lung metastases; IQR = interquartile range; mo = months; HR = hazards ratio; CI = confidence interval; NA = not available; NOS = not otherwise specified; Surg (pri) = surgical treatments of primary site.

## DISCUSSION

To the best of our knowledge, this investigation was the largest population-based study to estimate the prevalence, risk and prognostic factors for initial lung metastases in cases of high-grade osteosarcoma. Based on our results, lung metastases were found in 16.9% of the high-grade osteosarcoma patients at the initial diagnosis. The prevalence of initial lung metastases estimated in the present study was less than was seen in another study based on a single-center database, in which the prevalence was 29.5%.^11^

Because of the deleterious effect on survival generated through lung metastases among high-grade osteosarcoma patients, a predictive system for determining whether high-grade osteosarcoma patients have lung metastases and/or for choosing radiographic scanning needs to be delineated. Our results suggested that high-grade osteosarcoma patients were at significantly higher risk of developing lung metastases at diagnosis if their osteosarcomas were characterized by the primary tumor in an axial location, greater primary tumor size, higher N stage and presence of bone metastases or brain metastases. Thus, radiographic scanning ­and/­or further screening should be considered at diagnosis for high-risk high-grade osteosarcoma patients. We therefore recommend that physicians should construct risk assessment tools using the aforementioned risk factors and should provide different screening strategies for patients with different risk levels.

In terms of prognostic determinants, with three age groups analyzed among our osteosarcoma patients with lung metastases, multivariable Cox regression analyses showed that younger age (< 60 years) was one of the favorable prognostic factors among high-grade osteosarcoma patients with lung metastases. Our results were further strengthened by many previous studies that have revealed that a correlation exists between increasing age and poorer prognosis among osteosarcoma patients.^15^^,^^16^^,^^17^^,^^18^ Thus, more attention needs to be paid to elderly high-grade osteosarcoma patients with lung metastases.

In addition, the results showed that surgical treatment was a factor that favored greater survival among high-grade osteosarcoma patients with lung metastases. Accordingly, we would recommend aggressive surgical removal of the primary site tumor, in order to improve the survival of high-grade osteosarcoma patients with lung metastases. However, no information on the detailed surgical approaches adopted among osteosarcoma patients was recorded in the public SEER database.^19^ Therefore, no comparison of surgical approaches can be made, and surgical subgroup analyses cannot be conducted. Thus, we are unable to accurately recommend the type of surgery that should be used for treating osteosarcoma patients with lung metastases. Hence, further studies should be conducted in order to confirm the results in the future.

Inevitably, there are some limitations in the present study. Firstly, the SEER database does not gather information about local recurrence or metastases during the follow-up, which may affect the prognosis. Secondly, the detailed diagnostic approach for determining situations of an initial lung metastases and the surgical details of treatments implemented among osteosarcoma patients were not recorded in the public SEER database. Accordingly, no comparison of diagnostic approaches could be made, and surgical subgroup analyses could not be undertaken. Thirdly, among the osteosarcoma patients with lung metastases, asymptomatic cases and metachronous lung metastases cases were not recorded in the public SEER database. Hence, the real occurrence rate of lung metastases among osteosarcoma patients may have been underestimated. Lastly, due to the limited sample size, we could not maintain enough statistical power to get a more stable result and, consequently, further studies are needed in order to confirm these results.

The prevalence of initial lung metastases among our high-grade osteosarcoma patients was 16.9%. High-grade osteosarcoma patients with the primary tumor in an axial location, greater primary tumor size, higher N stage and presence of bone metastases or brain metastases were more likely to have lung metastases at diagnosis. Younger patients (< 60 years) and surgical treatment were factors that favored better survival among high-grade osteosarcoma patients with lung metastases.

## CONCLUSIONS

The associated factors, including primary tumor in an axial location, greater primary tumor size, higher lymph node stage and presence of bone metastases or brain metastases, were significantly correlated with lung metastases that were detected through screening among high-grade osteosarcoma patients. These factors can potentially be used for lung metastasis screening. The elderly age group (≥ 60 years) was found to be significantly correlated with poor overall survival among the high-grade osteosarcoma patients with lung metastases. To improve the survival of osteosarcoma patients with lung metastases, aggressive surgery on the primary tumor site should be encouraged.
